# Fibrinolytic Compounds Isolated from a Brown Alga, *Sargassum fulvellum*

**DOI:** 10.3390/md7020085

**Published:** 2009-04-09

**Authors:** Wenhui Wu, Keiji Hasumi, Hui Peng, Xianwen Hu, Xichang Wang, Bin Bao

**Affiliations:** 1 Department of Marine Biopharmacology, Shanghai Ocean University, Shanghai 201306, P.R. China; E-mail: whwu@shou.edu.cn; xcwang@shou.edu.cn; bbao@shou.edu.cn; 2 Department of Applied Biological Science, Tokyo University of Agriculture and Technology, Tokyo 1838509, Japan; E-mail: hasumi@cc.tuat.ac.jp; 3 Department of Chemistry, The University of Auckland, Auckland 1142, New Zealand; E-mail: h.peng@auckland.ac.nz; 4 Institute of Biotechnology, Beijing 100071, P. R. China; E-mail: hu.xianwen@tsinghua.org.cn

**Keywords:** fibrinolytic activity, NMR, glucopyranosyldiacylglycerol, structure determination, Sargassum fulvellum

## Abstract

Two of bioactive natural products were founded in a brown alga, *Sargassum fulvellum*. After isolation and purification, the molecular structures of these two products were investigated by NMR spectroscopy and GC-mass spectroscopy. The two compounds were identified to be *1*-*O*-palmitoyl-*2*-*O*-oleoyl-*3*-*O*-(α-D-glucopyranosyl) –glycerol (POGG) and *1*-*O*-myristoyl-*2*-*O*-oleoyl-*3*-*O*-(α-D-glucopyranosyl) – glycerol (MOGG) which were obtained from *Sargassum fulvellum* for the first time. POGG and MOGG showed fibrinolytic activity in the reaction system of pro-u-PA and plasminogen.

## 1. Introduction

Algae living in a unique marine environment are more and more important in drug development because they metabolize many rare compounds via unknown pathway [1]. A number of studies illustrated that algae were important resource of natural drug development. For examples, the red alga *Sphaerococcus coronopifolius* showed antibacterial activity [2]; the green alga *Ulva lactuca* contained an anti-inflammatory compound [3]. *Portieria hornemannii* (Lyngbye) P.C. Silva was found to be a novel source of cytotoxic penta halogenated monoterpene, halomon, which has been selected for preclinical drug development since this compound shows toxicity to brain, renal and colon tumor cell-lines and preliminary evaluations *in vivo* have been encouraging [4]. *Ulva fasciata* produced a novel sphingosine derivative which has antiviral activity *in vivo* [5]. A cytotoxic metabolite, stypoldione, which inhibited microtubule polymerization and thereby prevented mitotic spindle formation, has been isolated from tropical brown alga, *Stypopodium zonale* [6]. An iodinated novel nucleoside has been isolated from *Hypnea valitiae*, which is a potent and specific inhibitor of adenosine kinase. It can be used in the studies of adenosine receptors in a variety of systems, and in studies on nucleotide metabolism and regulation [7]. *Sargassum carpophyllum* from the South China Sea is the source of two new bioactive sterols. These sterols induced morphological abnormality in the plant pathogenic fungus *Pyricularia oryzae*; also exhibited cytotoxic activity against several cultured cancer cell lines [8]. There are many algae that can convert simple polyunsaturated fatty acids, such as arachidonic acids, into complex eicosanoids and related oxylipins [9]. This conversation is very important because derivatives of arachidonic acids play role in maintaining homeostasis in mammalian systems and aberrant production of metabolites of this class occurs in diseases such as psoriasis, asthma, arteriosclerosis, heart disease, ulcers and cancer [7].

The perennial brown alga *Sargassum fulvellum* has a wide distribution in the East China Sea. A polysaccharide with antitumor activity from *S. fulvellum* was studied [10] in early time. Also, sulfated polysaccharides isolated from *S. fulvellum* were found to have antioxidant activity [11]. Kang et al. reported [12] that solvent extracts from *S. fulvellum* has antipyretic, analgesic, and anti-inflammatory activities in mice.

Researchers have found a lot of compounds from algae including glycosphingolipids, glycoglycerolipids, polyphenol phosphate glycoside and steryl glycoside. Among them, glucosyldiacylglycerol has been reported to have a few biological effects, including significant fibrinolysis [13, 14], specific inhibition of tumor [15], and increased the cloning activation of phosphatidylserine decarboxylase in both the *Bacillus subtilis* null *pss* and *psd* mutants [16]. In this work, two of natural glucosyldiacylglycerol were isolated from a brown alga *S. fulvellum* using repeated chromatography with an open column of silica gel, and their structures and biological activities were investigated.

## 2. Results and discussion

*S. fulvellum* was selected by a screening of approximately 700 of plant extracts for compounds that enhance reciprocal activation of pro-u-PA and plasminogen. Specimen of *S. fulvellum* was collected from the East Sea in China and dried in natural environment. The dried samples were extracted by the mixture of methanol and chloroform. After removing the organic solvent, the residual was redissolved in chloroform and precipitated by the addition of acetone. The precipitate showing fibrinolytic activity was fractionated by repeated chromatography with an open column of silica gel and eluted with methanol and chloroform (proportion=3:7), to give compound 1 and compound 2. The yields of compound 1 and compound 2 from 200 g of brown alga were 25 mg and 15 mg, respectively.

**Figure 1:**
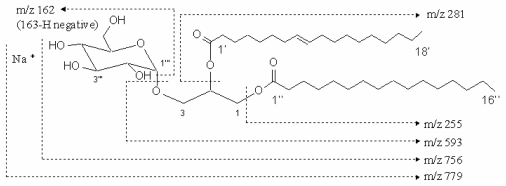
The molecular weight and fragment of compound 1. The molecular weight of compound 1 was m/z 779 with sodium ion and the fragments were m/z 593, 281, 255 and 162.

The compound 1 was colorless. Its infrared spectrum presented characteristic absorption bands at 2920 cm^−1^, 2852 cm^−1^, 1742 cm^−1^, 1219 cm^−1^, 1171 cm^−1^, 1035 cm^−1^, and 771cm^−1^. The positive-ion fast atom bombardment mass of the compound 1 gave the molecular weight with sodium ion at m/z 779 [M+Na]^+^ and fragment ions at 594 [M-163 (hexose moiety)+H]^+^, 256 [M-501 (163: hexose moiety, 281: oleoyl moiety, 57: glycerol moiety)+H]^+^, 282 [M-475 (163: hexose moiety, 255: palmitoyl moiety, 57: glycerol moiety) +H]^+^ by an assistant of trimethanolamine (Fig. 1). Using a negative-ion fast atom bombardment mode, other fragment ions were found at 162 [M-593 (281: oleoyl moiety, 255: palmitoyl moiety, 57: glycerol moiety)-H]^−^. These fragment ions suggested the molecule of this compound contains three parts, hexose unit and two fatty acyl moieties from single dimensional spectrum. The molecular formula was deduced to be C_43_H_80_O_10_ based on the fast atom bombardment masses, ^13^C and ^1^H NMR spectra.

The existence of two fatty acid residues in the molecular structure of compound 1 were also identified from the wrap over ^1^H and ^13^C NMR signals in the range of δ1.23~1.30 and δ28.30~28.97 respectively. Methanolysis of the compound 1 yielded two methylated fatty acid, methyl palmitate and methyl oleate, which were identified by comparing the GC-MS results with those of authentic standards [17].

**Figure 2:**
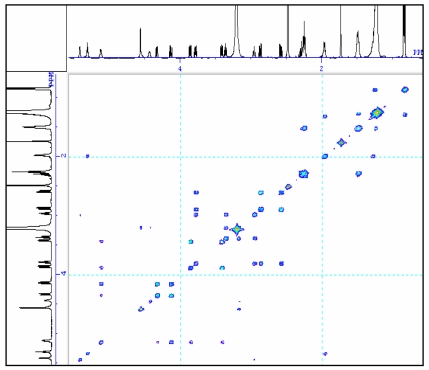
The ^1^H-^1^H COSY spectrum of the compound in DMSO-d_6_.

To further understand the molecular structure of compound 1, Table 1 gives the assignments of methine, methylene, methyl and quaternary carbon resonances obtained from ^13^C spectrum and ^13^C DEPT spectrum (90° and 135°). The chemical shifts of methine carbons in the sugar moiety appeared at 98.3 ppm, 71.6 ppm, 72.8 ppm, 74.4 ppm, 68.4 ppm and 54.9 ppm in ^13^C spectrum which could be assigned to C-1‴, C-2‴, C-3‴, C-4‴, C-5‴, and C-6‴, respectively. The spectral assignment of carbon resonances and proton resonances in methine of hexose was decided from HMQC (table 1) spectrum. In the ^1^H spectrum, the signal of the anomeric proton in the sugar moiety appeared at δ4.56 (d, *J* = 3.7), which showed a 1, 2-diequatorial relationship and indicated that the sugar moiety was α-configuration at C1 [18, 19]. The two dimensional correlation of the adjoining proton resonances was investigated by ^1^H-^1^H COSY. The correlation of neighbouring C-ring protons in hexose was found by corresponding cross peaks in the ^1^H-^1^H COSY spectrum (fig. 2). ^1^H NMR coupling constants and ^13^C NMR chemical shifts suggested that the hexose was glucose, and its J_1‴, 2‴_ (3.7 Hz, diaxial), J_2‴, 3‴_ (9.2 Hz, diaxial), J_3‴, 4‴_ (9.2 Hz, diaxial), and J_4‴, 5‴_ (9.6 Hz, diaxial) values in the ^1^H NMR spectrum proposed that the hexose of compound 1 should be α-D-glucopyranosyl [20]. The structure of glucose was further confirmed by the cross peaks of H-1‴ (δ_H_ 4.56)/H-2‴ (δ_H_ 3.18), H-2‴/H-3‴ (δ_H_ 3.36), H-3‴/H-4‴ (δ_H_ 2.94), H-4‴/H-5‴ (δ_H_ 3.78), H-5‴/H-6‴ (δ_H_ 2.58, 2.57) in ^1^H-^1^H COSY spectrum.

C-1, C-2, and C-3 were attributed to glycerol moiety, which were also deduced from two dimensional resonances of HMQC and ^1^H-^1^H COSY. The signals of C-1, C-2, and C-3 in single dimension carbon spectrum on middle field has coupling proton signals of 4.34 ppm (C-1), 4.21 ppm (C-1), 5.12 ppm (C-2), 3.87 ppm (C-3), and 3.41 ppm (C-3), respectively. The protons were acknowledged to adjoining oxygen atom because of its high magnetic field.

**Figure 3:**
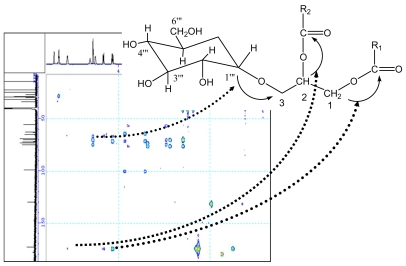
Important HMBC correlation for compound 1.

The glycosidic linkage between the glucose and glycerol was determined by glycosidation shift in ^13^C-NMR and HMBC spectra, the chemical shift of C-1‴ in glucose unit was at δ98.3. Its proton was obviously correlated with the signal of C-3 in HMBC spectrum. In HMBC spectrum, two correlations were observed between the proton signal at δ4.21 (dd, *J* = 2.6, 12.1) or δ5.12 and the quaternary carbon signal at δ172.2 or δ172.4, suggesting the glycerol moiety was attached to C-1′ or C-1″ of the fatty moiety. The carbons and protons resonances of fatty residue moieties possess correlation, resulting in many cross peaks in ^1^H-^1^H COSY (Fig. 2) and HMBC spectrum (Fig. 3).

In the spectrum of positive-ion fast atom bombardment mass, palmitoyl moiety of ion peak at m/z 256 was most notable at 100% of fragment strength, suggesting it was strongly bombarded by electronic flow due to weak electronic shielding effect. Therefore, palmitoyl moiety was determined at C-1 because the electronic shielding role of C-1 was weaker than those of C-2 and C-3 as shown in the ^13^C spectrum. Further, the oleoyl moiety was speculated at C-2.

Based on the above analysis of NMR data and mass spectra, the structure of the compound 1 was established to be *1* – *O* – palmitoyl – *2* – *O* – oleoyl – *3* – *O* – (α - D – glucopyranosyl) – glycerol (POGG).

The pure compound 2 was also colorless. Its rate of flow (Rf = 0.30) was smaller than that of compound 1 (Rf = 0.48) when it was eluted using chloroform and MeOH (proportion=3:7) including 1% of acetic acid (V/V). Its characteristic absorption bands in infrared spectrum were similar to compound 1. The positive-ion fast atom bombardment mass of the compound 2 gave the molecular weight with sodium ion at m/z 718 [M+Na]^+^ and mainly fragment ions at 212, which was identified to be myristoyl moiety by GC-MS. Table 2 presents the NMR data for compound 2. By the similar analysis as compound 1, the compound 2 was determinated to be *1* – *O* – myristoyl – *2* – *O* – oleoyl – *3* – (α - D – glucopyranosyl) – glycerol (MOGG).

**Figure 4:**
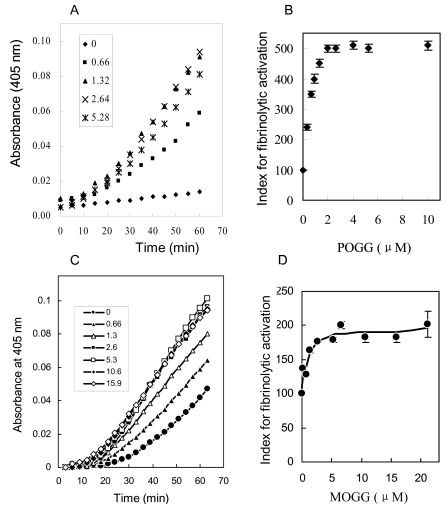
Effect of POGG and MOGG on reciprocal activation of pro-u-PA and plasminogen.

The fibrinolytic activities of compound 1 and 2 were investigated by the reciprocal activation of single chain urokinase-type plasminogen activator (pro-u-PA) and plasminogen. The addition of POGG (0.66–15.9 μM) or MOGG (0.66–5.28 μM) shortened the initiation time of pro-u-PA activation and increased the rate of activation *in vitro* (Fig. 4A, 4C), respectively. The dose-response curves were hyperbolic, and half-maximal effect were obtained approximately at 0.85μM of POGG (Fig. 4 B) and 1.3 μM (Fig. 4 D). These results illustrated that POGG and MOGG showed fibrinolytic activity in the reaction system of pro-u-PA and plasminogen, and MOGG exhibited weaker fibrinolytic activity than POGG.

Pro-u-PA and plasminogen were incubated with the indicated concentrations of POGG and MOGG in the presence of Spectrozyme UK to determine reciprocal activation of pro-u-PA. Time course of the POGG and MOGG effect is shown in A, C and dose-response curve in B, D, respectively, in which the index for fibrinolytic activation is given in ordinate as percent of control value.

## 3. Conclusions

In this work, two of glucopyranosyldiacylglycerols, POGG and MOGG were isolated from the brown alga *S. fulvellum*, and there molecular structures were determined by NMR spectroscopy and mass spectroscopy. The extracts of *S. fulvellum* have been reported to have many biological roles, including antipyretic, analgesic, anti-inflammatory [12], antitumor [10] and antioxidant [11]. According to our knowledge, this is the first time to isolate MOGG from marine lives and discover that POGG and MOGG enhanced reciprocal activation of pro-u-PA and plasminogen *in vitro*.

## 4. Experimental section

### 4.1 General experimental procedures

Fast atom bombardment mass were determined with an assistant of trimethanolamine on a SX-102A mass spectrometer (JEOL USA Inc.). The infrared spectra were measured on a JIR-WINSPEC 50 FT-IR spectrometer (JEOL, Tokyo, Japan). ^1^H-NMR (600 MHz), ^13^C-NMR (150 MHz), and DEPT spectra were recorded on a JNM alpha 600 FT NMR spectrometer (JEOL, Tokyo, Japan) in dimethylsolfate-*d*_6_ (DMSO-*d*_6_), and chemical shifts are given in the δ (ppm) scale, with coupling constants (*J*) in Hz with tetramethylsilane as a contrast standard. Procoated Kieselgel 60 F_254_ cards (thickness 0.2 mm, Merck, Germany, No. 5450) were used to analysis of purification for thin layer chromatography using the solvent system of chloroform-methanol-acetic acid. Spot detection was conducted by spraying iodine vapor at room temperature.

The heteronuclear multiple quantum coherence (HMQC) experiment was carried out at 600.05 MHz with a sweep width of 4168.0 Hz and 2995.8 Hz. A 1.2-seconds relaxation delay was used and 64 transients were performed for each t_1_ value.

The ^1^H-^1^H correlation spectroscopy experiment was carried out at 600.05 MHz with a sweep width of 4164.3 Hz and 2989.5 Hz. A 0.83-second relaxation delay was used and 16 transients were accumulated for each t_1_ value.

The ^1^H detected multiple bound connectivity experiment was carried out at 600.05 Hz with a sweep width of 4165.4 and 2995.8 Hz in F_1_ and F_2_, respectively. The data set 512 points, and the mixing time 60 msec. The spectrum was recorded in the absorption mode.

### 4.2 Isolation of biophysiological substance

The compounds were extracted with chloroform-methanol (2:1) from *S. fulvellum* and isolated by repeated chromatography with silica gel columns. Briefly, dried *S. fulvellum* (200 g) was extracted twice with 900 ml of chloroform-methanol (2:1) and once with 900 ml of chloroform-methanol (1:2), and the organic extracts was evaporated to dryness, giving 15.1 g of oily residue. The residue was redissolved in 15.1 ml of chloroform, followed by the addition of 300 ml of acetone. The precipitate was collected by centrifugation. The precipitate (500 mg) was further fractionated by repeated chromatography with silica gel columns using solvent systems of chloroform-methanol and chloroform-methanol containing 1% acetic acid. Active component (15.1 mg) was recovered in a chloroform fraction in the final step.

### 4.3 Methanolysis of the compound

The compound 1 (3 mg) were dissolved in 1.5 ml of methanol solution (82%) containing 0.9 N of HCl. The solution was heated to 70° for 18 hours [21, 22]. Then the solvent was evaporated to 0.1 ml under reduced pressure at 40°, followed by the extraction with hexane to yield an organic layer, which was purified using silica gel chromatography with elution of chloroform. The sample was dissolved in methanol for analysis by a JMS-GC Mate II (JEOL, Peabody, MA). A silica capillary GC column (30m 0.25mm, Agilent Technologies, Inc.) was used, and the column oven temperature was kept at 80 °C for 1 min after sample injection and then increased at a rate of 32° per min up to 150°, subsequently at a rate of 4° per min up to 250°, the flow rate of helium carrier gas was 1 ml/min. Identification of the peaks was accomplished by comparing the relative retention time and the mass spectra with those of standard fatty methyl esters.

### 4.4 Analysis of fibrinolytic activity

The fibrinolytic activities were determined in 50 μl of TBS/BSA (50 mM Tris-HCl, 100 mM NaCl and 2 mg/ml bovine serum albumin, pH 7.4) at 37°C using a round-bottomed 96-well plate. The release of *p-*nitoaniline was measured serially for up to 120 min at 405 nm. The concentrations of pro-u-PA, plasminogen and Spectrozyme UK were as 20 nM, 5 nM and 100 μM, respectively.

## Figures and Tables

**Scheme 1:**
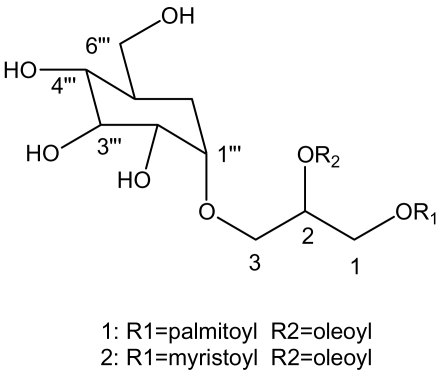
Structures of compound 1 and 2.

**Table 1:** The ^1^H and ^13^C NMR data of compound 1 from *Sargassum fulvellum*.

No.	δ_C_	δ_H_
1	62.5	4.34 (1H, dd, *J* = 2.6, 12.1), 4.21 (1H, dd, *J* = 2.6, 12.1)
2	69.7	5.12 (1H, m)
3	64.7	3.87 (1H, dd, J = 5.8, 10.6), 3.41 (1H, dd, J1=5.5, 10.6)
1‴	98.3	4.56 (1H, d, *J* = 3.7)
2‴	71.6	3.18 (1H, dd, *J* = 4.4, 9.2)
3‴	72.8	3.36 (1H, t, *J* = 9.2)
4‴	74.4	2.94 (1H, t, *J* = 9.2)
5‴	68.4	3.78 (1H, ddd, *J* = 5.1, 5.5, 9.5)
6‴	54.9	2.58 (1H, dd, J = 4.8, 13.9), 2.57 (1H, dd, J = 6.2, 13.9)
1′, 1″	172.4, 172.2	
2′, 2″	33.5, 33.3	2.26 (4H, m)
3′, 3″	24.3	1.49 (4H, m)
4′ – 7′, 12′ – 15′, 4″ – 13″	28.3 – 28.9	1.2 – 1.3 (m)
8′, 11′	26.5, 26.4	1.97 (4H, m)
9′, 10′	129.5, 129.4	5.04 (2H, t, *J* = 4.8)
16′, 14″	31.2	1.2 – 1.3 (m)
17′, 15″	21.9	1.2 – 1.3 (m)
18′, 16″	13.7	0.84 (6H, t, *J* = 6.8)

The chemival shift is relative to DMSO - *d*_6_ (δ_C_ 39.5 ppm; δ_H_ 2.49 ppm).

The coupling constant (*J*) is given in Hz.

The assignments of the two fatty acyl moieties are tentative and concomitantly interchangeable.

**Table 2:** The ^1^H and ^13^C NMR data of compound 2 from *Sargassum fulvellum*.

No.	δ_C_	δ_H_
1	62.6	4.09 (1H, dd, *J* = 7.4, 11.4), 4.30 (1H, d, *J* = 11.8)
2	69.7	5.10 (1H, d, *J* = 5.0)
3	64.6	3.36 (1H, dd, *J* = 5.9, 10.3), 3.85(1H, dd, *J* = 5.8, 10.5)
1‴	98.3	4.54 (1H, d, *J* = 3.5)
2‴	71.6	3.15 (1H, m)
3‴	72.9	3.32 (1H, t, *J* = 8.9)
4‴	74.2	2.88 (1H, m)
5‴	68.5	3.74 (1H, m)
6‴	54.5	2.52 (1H, dd, *J* = 6.8, 12.5), 2.88 (1H, m)
1′, 1″	172.3, 172.4	
2′, 2″	33.4, 33.6	2.22 (2H, t, *J* = 7.1), 2.26(2H, m)
3′, 3″	24.4	1.46 (4H, dd, *J* = 6.7, 13.4)
4″ – 7″	28.4–29.1	1.2 – 1.3 (m)
8″, 11″	26.5, 26.6	1.2 – 1.3 (m), 1.94 (2H, m)
9″, 10″	129.4, 129.5	5.27 (1H, t, *J* = 4.5), 5.35 (1H, d, *J* = 2.5)
12″ – 15″	28.4–29.1	1.2 – 1.3 (m)
4′ – 11′	28.4–29.1	1.2 – 1.3 (m)
16″, 12′	31.3	1.2 – 1.3 (m)
17″, 13′	22.1	1.2 – 1.3 (m)
18″, 14′	13.9	0.81 (6H, t, *J* = 6.7)

The chemical shift is relative to DMSO-*d*_6_ (δ_C_=39.5 ppm; δ_H_=2.49 ppm).

The coupling constant (*J*) is given in Hz.

The assignments of tow fatty acyl material are tentative and concomitantly interchangeable.

## Abbreviations

COSY: Correlation spectroscopy
HMQC: ^1^H detected multiple quantum coherence
HMBC: ^1^H detected multiple bound connectivity
DEPT: Distorsionless enhancenment by polarization transfer
pro-u-PA: single chain urokinase-type plasminogen activator
